# Caregivers' Experience of Decision-Making regarding Diagnostic Assessment following Cognitive Screening of Older Adults

**DOI:** 10.1155/2018/8352816

**Published:** 2018-12-02

**Authors:** Jamie J. Y. Lee, Joanna Barlas, Claire L. Thompson, Yan Hong Dong

**Affiliations:** ^1^School of Psychology, James Cook University, Singapore; ^2^School of Psychology, Central Queensland University, Rockhampton, Australia; ^3^Centre for Healthy Brain Ageing (CHeBA) and Dementia Collaborative Research Centre—Assessment and Better Care, School of Psychiatry, UNSW Medicine, The University of New South Wales, Sydney, Australia

## Abstract

Targeted screening for dementia among older adults in primary healthcare has potential benefits such as better clinical outcomes and the opportunity to access services. Cognitive screening can be followed up by further diagnostic assessment to determine a diagnosis of dementia. Unfortunately, the rates of accepting further diagnostic assessment following cognitive screening are low. The objective of this study was to explore the caregivers' decision-making process regarding uptake of diagnostic assessment following positive screening results. A qualitative design was employed, and interpretative phenomenological analysis was used to analyze the data. Three major themes in caregiver decision-making were identified: gathering information, protecting the patient, and balancing obligation and convenience in caregiving. These findings suggest that the decision-making process involved effort to process information through observations of the patient and that caregivers emphasized quality of life.

## 1. Introduction

Dementia is a general term that describes cognitive impairment severe enough to impair daily functioning and results in significant cognitive decline and disability among older adults. It is a public health priority that has high economic costs and contributes to caregiver burden and reduced quality of life for both patients and caregivers [1]. Prevalence rates indicate that 47.5 million people worldwide currently have dementia, with a projection that 75.6 million people by 2030 and 135.5 million people by 2050 will have the disease [1]. The prevalence of dementia among older adults aged 60 and above in Singapore is 10%. In 2012, 28,000 older adults over 60 in Singapore were diagnosed with dementia; this figure is projected to increase to 80,000 by 2030 [2]. Despite high prevalence rates, dementia is often undetected by physicians in primary healthcare [3, 4].

Targeted screening of older adults in primary healthcare has the potential benefits of higher detection rates and early commencement of treatment. Early treatment results in better clinical outcomes and more opportunities for access to services and advanced care planning [5–7]. Patients with a positive screen result are asked to follow up with further diagnostic assessment which could include neuropsychological testing, laboratory testing, and physical examinations [8, 9]. Unfortunately, there are low rates of follow-up with further diagnostic assessment following screening [10, 11], and without sufficient follow-up, patients are exposed to the risks associated with a false-positive or false-negative screening result. False-positive results may lead to unnecessary psychological distress and stigma associated with dementia [7, 12]. False-negative results are problematic as the benefits of early treatment are denied, and undetected cognitive impairment may lead to difficulties undertaking activities of daily living that place the older adult at risk of harm [13]. It is therefore important for patients to attend further diagnostic assessment following a positive screen. A previous research identified the low rates of follow-up after cognitive screening in Singapore [14]. The current study sheds light on low follow-up rates through exploration of caregiver decision-making regarding the uptake of further diagnostic assessment.

Individuals with dementia may lose decision-making capacity [15], and caregivers often play an increasingly large role in decision-making regarding medical, legal, and financial matters [16]. Caregiver and patient participation in decision-making ranges from patients' direct involvement [17–19] to patients' refusal to participate in decision-making to avoid fears about future cognitive decline [20, 21]. Caregivers play a bigger role in substitute decision-making; this usually occurs when patients have greater cognitive impairment and reduced decision-making capacity [22]. Early detection of cognitive impairment facilitates decisions regarding use of dementia care services and planning of caregiver duties, which benefits both people with dementia and their caregivers [6]. Furthermore, early detection allows caregivers to access psychosocial support such as support groups and caregiver training programs that have been found to improve morale and ameliorate stress [23, 24].

Fowler et al. [25] examined the association between attitudes and uptake of screening among patients and found that patients' attitudes about benefits of dementia screening have an impact on the acceptance of dementia screening. Persons with dementia have cited benefits to knowing their diagnosis such as having the opportunity to address memory problems, settle financial and legal matters, and understand behavioural changes [26]. Several other factors such as perceived benefits of dementia screening, age, need for future planning in the event of dementia, and gender have been associated with acceptance of dementia screening in primary care [27, 28].

Research evidence sheds some light on the reasons why people reject diagnostic assessment or disclosure of a dementia diagnosis [7, 29, 30]. Boustani et al. [29] found that barriers to screening identified by two groups of participants, those with dementia caregiving experience and those without, were emotional suffering by the family, loss of driving privileges, and becoming depressed. Research on the characteristics of patients who received a positive screen for dementia but refused further diagnostic assessment indicated that greater perceived stigma and living alone were associated with greater odds for refusing diagnostic assessment [31].

Stigma of having a dementia diagnosis has resulted from the disease's association with negative outcomes like a lack of cure, loss of dignity and autonomy, and being excluded and discriminated against by family and healthcare professionals [32]. Stigma has influenced the minimization and concealment of symptoms that leads to late presentation to services, delays in obtaining a diagnosis after presentation due to therapeutic nihilism (belief that no effective intervention or treatment is available for dementia) and pessimism about prognosis, and medical professionals' reluctance to provide a diagnosis of dementia [32, 33].

Lee et al. [34] surveyed the caregivers of participants who had completed screening and preliminary neuropsychological testing to examine uptake of further diagnostic evaluation and the predictors of uptake. Of 140 participants, 33 were identified at screening to have moderate or severe risk of cognitive impairment and were asked to follow up with further diagnostic assessment [34]. The current study is a follow-up to the previous study [34] and explores how caregivers participated in the decision-making for further diagnostic assessment. This is the first study to explore the subjective experience of decision-making following cognitive screening of older adults using a qualitative methodology.

## 2. Materials and Methods

This study employed a qualitative design using interpretative phenomenological analysis (IPA). IPA is a qualitative approach to examine how individuals perceive, reflect on, and make sense of their experiences [35]. Notably, no research has examined the caregivers' decision-making process for patients to follow up with diagnostic assessment following screening, and therefore, the IPA approach was chosen to explore the subjective experience of decision-making. IPA emphasizes in-depth analysis of individual perspectives [36] and recommends a sample size of three to six participants [37].

Of 140 participants in a study by Lee et al. [34], 33 were identified to have moderate or severe risk of cognitive impairment and were asked to follow up with further diagnostic assessment [34]. The caregivers of these 33 participants were invited to participate in the current study. Six caregivers who consented were interviewed, and one caregiver's data were excluded from analysis because he was unable to provide coherent responses. The five remaining participants were all female and were adult children (one daughter-in-law and four daughters) who lived with and cared for the patient. The remaining caregivers of the original 27 people with moderate or severe risk of cognitive impairment declined the current study's invitation. The 27 who declined to participate had a wider variability of age (37 to 77, *M* = 57), gender (70.4% female), and relationship with patient (7.4% husband, 25.9% wife, 22.2% son, and 44.4% daughter) compared to those who participated. However, the mean age, female gender, and daughter/daughter-in-law relationship of the participants suggest they are typical of the wider sample from which they were recruited.

Data were collected via semistructured interviews with caregivers to explore the decision-making process regarding accepting further diagnostic assessment following screening. Interviews were guided by a list of questions such as “How did you and the care recipient decide to accept/decline further diagnostic assessment?” “What helped you during the decision-making?” and “Who was involved in this decision-making process?”

Participant demographics are displayed in Table 1.

Following ethical approval by the National University Hospital and a university's ethics committees, interviews that lasted approximately 40 minutes were conducted in English at caregivers' homes. A member of the research team undertook analysis of the data obtained from verbatim transcripts of interviews.

Using an idiographic IPA approach to data analysis, descriptive (focusing on the content of the narrative), linguistic (focusing on the use of language), and conceptual (focusing on understanding the narrative at an abstract level) comments were identified [35]. The first author mapped interrelationships, connections, and patterns among these comments, and emerging themes were examined. A list of master themes and subthemes was then generated. This process was repeated for all five transcripts, and cross-case analysis was subsequently conducted. Themes from each transcript were analyzed and compared for convergence and divergence, leading to a final list of master themes and subthemes.

An independent audit was conducted by the second author to affirm the validity of research findings. This process examined comments and themes identified, offered additional interpretation, and improved the validity of research findings by ensuring that interpretations were grounded in the data [35].

## 3. Results

Major themes derived from the current analysis included gathering information, protecting the patient, and balancing obligation and convenience in caregiving. Discussion of results includes a description of the master themes and subthemes (Table 2).

### 3.1. Theme 1: Gathering Information

 Following recommendation from the hospital to undertake further diagnostic assessment, caregivers described a process of gathering information about the patient's current functional status primarily through observation of the patient. They then appeared to process this information by making comparisons between the patient's previous and current functioning. All caregivers engaged in this process of observing the patient and making comparisons irrespective of whether the decision was to accept or refuse diagnostic assessment.

### 3.1.1. Observing the Patient

Caregivers monitored patients' functional abilities, memory, and social activities, comparing the patient to others and taking note of any decline. The process of observing and acknowledging the relative's cognitive problems appeared to cause caregivers sadness and anxiety. One participant described watching her mother more closely after the initial screening process and was teary-eyed while talking about the changes she observed. Her anxiety over the patient appeared to contribute to her decision to accept further diagnostic assessment:

So I, staying with her, I monitor her daily routine, behaviour you see. So I found that she [pause] there were some changes lah, like normally in the evening she will take a shower, she will come down, she will go for her prayer. But I've realised ah, when she go up she never come down. And then she take so long to come down, so I was thinking what happened. [Participant 2]

Caregivers reflected on their observations of patients during preliminary neuropsychological testing, using that as a means of gauging patients' ability to cope with further diagnostic assessment. The patient's emotional state was an important factor that informed the decision-making process. In one case, the fear and grief observed during this preliminary neuropsychological testing led a caregiver to refuse further assessment:

But I didn't realise that the test would be so elaborate. That it would cause her so much grief. So you know, the thought of someone coming to do these tests, had caused her so much fear. [Participant 1]

### 3.1.2. Making Comparisons

Caregivers made comparisons between the patient's past and present functioning and the patient's physical versus cognitive difficulties. Comparing the familiar to the unfamiliar appeared to be a means of gauging the patient's deterioration to inform the decision. One caregiver talked about how the patient's abilities for activities of daily living had deteriorated, using this functional deterioration to justify a decision to follow up with diagnostic assessment:

And then instead of looking into the mirror and putting on her cream, she'll just put it on without looking and she'll go out with patches on her face, that kind of thing. It's so different from what she used to do. [Participant 3]

It was noted that caregivers tended to discuss patient's physical difficulties when asked about patient's cognitive difficulties. For example, a caregiver talked about the patient's refusal of a prior leg operation to assess the level of resistance the patient would have towards further diagnostic assessment. This also implies that the patient's consent and views regarding further testing were pertinent to the decision-making process. Refusal from the patient was understood in the context of the patient's character and respected even when it caused distress to the caregiver:

I just feel a little bit upset that if she really don't want to go for diagnosis, like maybe it get worse very soon then how to prevent… Because my mother, we know one, that's why my brother did not even want to talk to her. My brother say ‘leave it to her, if she want to go you bring, if she really refuse to go then forget it.' Yah, her character [is] that she think that she's alright. [Participant 4]

In their struggle to determine the extent of patients' cognitive impairment, caregivers talked about patients' cognitive decline in terms of what they were still capable of accomplishing rather than what abilities they had lost. This likely made it easier for the caregiver to acknowledge the cognitive decline and to discuss it openly. Focusing on patients' accomplishments appeared to facilitate the decision to refuse further assessment:

But as of this moment, she is still alright lah, not that serious lah. Occasionally, of course, when she talk, always repeating, still repeating, But other than that, like lunch, dinner or taking medicine, this kind of normal daily work, she still can manage lah. [Participant 4]

### 3.2. Theme 2: Protecting the Patient

 Caregivers' decisions appeared to stem from a position of wanting to protect the patient from perceived harm. Harm was perceived to come from either psychological distress associated with obtaining a dementia diagnosis or from the fatigue associated with changes in the patient's routine or environment during further assessment. As such, consideration of quality of life played a significant role in caregivers' protectiveness of patients. However, the definition of quality of life differed for those who declined versus those who accepted further assessment. Notably, caregivers who accepted diagnostic assessment differed from those who rejected in their perception of what they were protecting the patient from. For caregivers who rejected diagnostic assessment, quality of life represented maintenance of the status quo and avoidance of perceived stigma and distress. For those who accepted, quality of life was associated with access to treatment to slow cognitive decline that would otherwise progressively impair activities of daily living.

### 3.2.1. Protecting From Distress

Patients' apparent distress during preliminary neuropsychological testing elicited a protective stance from some caregivers. For example, one caregiver described her mother looking at her to stop the test. This caregiver appeared to assume the role of an advocate for her mother, where she was responsible for protecting her by refusing further diagnostic assessment:

Actually, she was the one who said ‘I don't want [testing] anymore… And when we talked about why [the tester] was coming, she said ‘I don't want anything anymore. It's time for me to go.' She didn't want to anymore… [During testing] she said, ‘Ah I got headache already' and then she was looking at me, because I sat next to her, she was looking at me to tell him to stop. [Participant 1]

Caregivers also reflected on the stigma of dementia that led to patients' fears of being abandoned and losing mental control. For some, this meant refusing further diagnostic assessment to avoid exposing the patient to personal distress and negative emotions associated with stigma. Participant 4 described her mother's emotional state when she was asked to attend further testing:

Very... Very angry lor. Like, like why you all think that I got problem? So we do not want to insist, since she think she is alright then later on she might think that wah, we treat her like got mental problem, so we just leave it. [Participant 4]

Other caregivers who recognized the same potential for stigma and distress but chose to proceed with further diagnostic assessment did so by being deliberately vague about the purpose of further assessment:

So we try to tell her we are bringing you to a doctor who is seeing old folks problem. We don't want to tell her ‘shi zhi zhen' [dementia in Mandarin]. Because we don't want to make her frighten also… she's always talking about who's having dementia, and who behaving like that. That kind of thing you see. She has this fear of that. So we also don't want to put her into that state of shock. [Participant 3]

They justified this lack of transparency as protecting the patient from the emotional impact of decision-making where the patient would have to consider the possibility that she had dementia.

### 3.2.2. Protecting From Futility

Caregivers' understanding of dementia was closely related to their differing reasons for protectiveness of the patient. Those who talked about a lack of cure for the disease and the futility of further treatment in view of the patient's age and deterioration chose to refuse further assessment. Caregivers appeared to gauge what the patient would want by reflecting on the type of end-of-life care they preferred for themselves. For example, a caregiver communicated a belief that prolonging life unnecessarily could lead to further impairment in her mother's physical and social functioning:

Yah. I feel that life to a certain extent is meant for a certain span. After that, you cannot expect to go on living a good life. Your quality of life will go down. I think to a certain extent, putting you on a respirator and things like that is stupid. Nature should take its course. [Participant 1]

Conversely, caregivers who accepted further assessment defined quality of life as slowing deterioration so that the patient preserved the ability for certain activities of daily living and retained a sense of self. In this way, this caregiver was protecting what she believed was important to her mother:

Dementia there's no cure, but you can stop the deterioration… In order to let her be herself, like before, you need some medical help… If you don't follow up, her quality of life will be down. [Participant 2]

### 3.3. Theme 3: Balancing Obligation and Convenience

 Caregivers described a tension between caring for patients and maximizing the convenience of caregiving. Considering further assessment, caregivers thought about both the duty to act in the best interests of the patient and practical considerations like the cost and convenience of further assessment.

### 3.3.1. Entrusted as Advocate

This subtheme reflected how caregivers perceived a duty to care as being entrusted with the patient's physical and emotional well-being and having the responsibility of representing the patient's views and opinions about assessment and treatment. Caregivers were familiar with patients' values, preferences, and priorities—often, patients preferred to focus on physical problems over cognitive problems in medical treatment. It is therefore likely that more time and resources were allocated to the treatment of physical ailments than investigating cognitive concerns. One participant described how her mother was more concerned with painful corns on her feet than possible cognitive decline, and prioritizing physical care within this family led to the decision to refuse further diagnostic assessment:

Things that are important to her–her corns, she has very painful corns that sometimes leads her to not being able to move and sometimes I can't even get an appointment at the polyclinics. And then it gets worse to walk… To her, these two things matter more than her mind. In her mind, she's fine. [Participant 1]

For one participant, filial piety was associated with gratitude towards her mother. This gratitude and filial piety may have underpinned the decision to refuse further diagnostic assessment via a desire to protect the patient from distress or perhaps a belief that a formal diagnosis is irrelevant to the duty of care; children will look after their elderly parents no matter what the circumstances:

Now my turn to take care of her. That time only, I got three daughters, Primary 5, Secondary 1 [and] Secondary 4. My mother still can walk a little bit. She helped me. I go work. She looked after my daughter. Since my mother like that, I have to take of my mother. [Participant 5]

For participants that rejected diagnostic assessment, duty of care to the patient encompassed taking on the role of decision-maker and was related to the extent the caregiver perceived that the patient lacked capacity for rational or informed decision-making. Caregivers appeared to feel the weight of responsibility associated with decision-making, likely due to a perception that the patient placed absolute trust in them as an advocate and caregiver. Participant 1 elicited her mother's views and used them as a basis to refuse further diagnostic assessment:

Because she knows that of the three children, it's me that cares for her interest. So whatever I'm doing, it's for her best interest. She never doubted it. Because after that I did ask her, did you want to go for more tests [further diagnostic assessment]? She said no, I don't want. [Participant 1]

Conversely, two out of five caregivers who accepted diagnostic assessment overrode patients' preference to reject diagnostic assessment. This was related to a similar duty of care, although in these cases the caregiver decided that the patient lacked capacity to make good decisions and therefore stepped in to make what appeared to be a “best interest” decision:

Because you know, at this point, she cannot make decision anymore. And she doesn't make very rational or she doesn't make good decisions rather… I would say that she leave a lot of the decision to see doctor with us. [Participant 3]

Even when overriding patient's preferences, participants were not transparent in explaining that further assessments were to diagnose dementia. The lack of transparency could stem from a desire to protect the patient (as per Theme 2) or to avoid negative reactions and resistance from the patient. This caregiver expressed frustration over not being able to communicate the purpose of further diagnostic assessment. This could also be due to not knowing how to communicate the purpose of further tests to her mother-in-law who suffered some cognitive impairment and had difficulty understanding information:

I want to tell her, but how to tell her? I think she doesn't understand. And then if she, she heard what I say, next minute she will forget what I say already. [Participant 2]

### 3.3.2. Easing Caregiver Burden

The burden of caregiving appeared to factor into caregivers' decisions. Caregivers' struggles to manage their own physical ailments, finances, and work schedule added to the burden of caregiving.

Caregivers described family discussions as either an obligatory part of caregiving or a means of receiving support to manage the burden of care. For instance, one participant described regular family discussions about caregiving arrangements as helpful, which may be particularly salient within families where the primary caregiver and the patient have different views about going for further diagnostic assessment:

For us, when we see something is wrong, sometimes I will call her [participant's sister]. Sometimes, she will just check with me if everything is ok, and then if I suspect something is wrong, we will just talk about it. It's actually regularly [referred to discussions with sister]. [Participant 3]

Caregivers considered various factors such as the cost of further assessments and the patient's difficulties with mobility. These factors increased the burden of care and contributed to the decision to refuse further assessment. Participant 1 communicated a desire to reduce the need to impose additional travel on her mother which led to a refusal of further diagnostic assessment:

You know, getting in and out of the car, so as much as possible, even now she doesn't want to go for her evening strolls in the wheel chair. She says it makes her giddy. So you can see that more movement is causing her to get this [giddy]. She gets like a vertigo attack, and she starts throwing up. So as much as possible I don't want much change in her life. [Participant 1]

Other caregivers considered how further diagnostic assessment and treatment could ease caregiving burden as slowing the rate of cognitive deterioration could enable better management of difficult daily behaviours. Participant 3 expressed that further assessment could highlight areas in the patient's life that required more assistance, thereby facilitating ease of caregiving:

*Because I know it will help us… It's better than we just, we try to look after her blindly and not know what's going on or what has gone wrong. It's better that we know exactly. Because this test will actually zero in right, which area and where she needs help. So we, we in fact we are happy to have such thing you see. We do*n'*t mind the inconvenience. Ultimately it will help her and it will help us as well. [Participant 3]*

## 4. Discussion

This study explored caregivers' experience in decision-making regarding patients' participation in further diagnostic assessment to confirm cognitive screening results for dementia. Caregivers described a process of monitoring the patient, drawing parallels, and making comparisons between the patient's cognitive difficulties and their physical ailments—this suggests an effort to process information leading up to a decision. Healthcare professionals advocating further testing may therefore find it helpful to discuss cognitive impairment in relation to physical problems as a means of facilitating caregivers and patients' understanding of dementia. Caregivers appeared to worry more about patients' physical health compared to cognitive health. Furthermore, cognitive problems appeared to receive more attention when they progress to a point which markedly increases caregiver burden. Hence, psychoeducation regarding how addressing cognitive impairment helps reduce caregiver burden could increase motivation for attending further diagnostic assessment.

While all caregivers considered patients' preferences, the caregivers in this sample reported that they played a bigger role in the decision-making than patients did. This could be due to patients' risk of moderate to severe cognitive impairment. While caregivers who rejected further testing highlighted that the patient's refusal significantly influenced the decision, those who overrode patients' preferences and opted for further tests considered the patient's inability to make informed and rational decisions.

There was a lack of transparency by caregivers about the purpose of testing as caregivers saw this as a way of protecting the patient from distress. This aligns with the previous research which showed that approximately 80% of a sample of caregivers (*n*=100) wanted to withhold a dementia diagnosis from patients due to fear of causing distress [37]. Medical practitioners who have withheld a diagnosis of probable Alzheimer's disease from patients have similarly revealed beliefs that patients would have reduced motivation and hope if they were informed of their diagnosis [38]. Issues such as the patient's right to know and the importance of being transparent with information appeared to be less salient for caregivers. This is at odds with a humanistic perspective of dementia that promotes the concept of personhood which states that recognition of the needs and rights of a patient with dementia enhances their well-being [39, 40].

Confucianism is a school of philosophy that has shaped family structures and behaviours in East Asia [41]. Values such as filial piety and interdependence that stem from the Confucian tradition [41] likely explain caregivers' keen interest in protecting the patient's well-being. Protecting a patient from distress by not providing full information could be an unspoken norm for caregivers in Singapore. Research has also shown that people from Asia use less open communication styles compared to European Americans [42]. It could then be that decision-making was further affected by a cultural norm where caregivers used less open communication styles and were less active in eliciting patient's preferences. Interestingly, the lack of open communication between caregivers and patients in the current study seems to have made decision-making easier for some participants because they were able to make decisions independently without the need to represent all views equally.

Caregivers' understanding of dementia influenced their protective stance over the patient. Therapeutic nihilism, or the belief that there is no available intervention for dementia, has been reported by doctors in primary healthcare [43, 44]. This perhaps contributed to some caregivers' desires to shield patients from psychological distress associated with the stigma and hopelessness of a diagnosis of dementia and the perceived futility of intervention.

Patients with dementia and their caregivers often focused on quality of life. While past research has discussed quality of life in end-of-life care decisions and assumed that there is a shared understanding of the meaning of quality of life [45], the current findings highlight that the definition of quality of life can vary among caregivers, thereby shaping decisions that lead to different outcomes. In our study, caregivers either strove to preserve quality of life by protecting from the distress of obtaining a diagnosis or strove to preserve quality of life by protecting from further decline through access to diagnosis and treatment. This is a central feature of the current results that highlights the need for healthcare professionals to facilitate caregivers' reflection on their understanding of quality of life, which would in turn influence caregiver involvement in further diagnostic assessment and treatment options.

Decision-making was made more difficult for these caregivers because of the guilt and sadness they experienced in this process. Efforts to minimize caregiving burden could contribute to caregiver guilt. Patients were resistant to diagnostic assessment as they associated dementia with going “crazy” and being abandoned. Patients' fears of being abandoned likely elicited guilt from caregivers. This is consistent with research on decisions about nursing home placements and end-of-life care being marked by caregivers' struggles with guilt and sadness [46, 47]. Additionally, the decision to accept diagnostic assessment draws caregivers closer to other difficult decisions such as residential care placement, which could exacerbate feelings of guilt and sadness.

The current findings support a recommendation for psychoeducation targeted at caregivers and patients to destigmatize dementia and provide accurate information on which to base decisions. Content might include information about the behavioural, psychological, and social changes that can occur as the disease progresses and how treatment might impact these changes. Psychoeducation might also address common negative perceptions of dementia to provide a more balanced and less condemnatory framework for understanding the disease. Caregivers and patients would also benefit from additional support from healthcare professionals during the decision-making process. Individualized meetings with a healthcare professional could help facilitate the expression of caregiver and patient views regarding further testing.

A limitation of the current study is that the current participants were all female within a limited age range and all in parent-daughter/daughter-in-law dyads; thus, no exploration of gender, age, or relationship differences in the decision-making process was possible. It is also important to note that this is a study of caregivers' decision-making: direct accounts of patients' experiences were not obtained. Future research could examine the experience of decision-making of a wider variety of caregiver demographics as well as older adults who are less cognitively impaired and able to provide reliable data.

## 5. Conclusions

Low rates of participation in diagnostic assessment following screening for cognitive impairment among older adults are a key concern. This study shows that caregivers played a significant role in determining uptake of further testing. Caregivers found this decision-making process difficult because of the guilt and sadness they experienced. Their decision was informed by their understanding of dementia and the disease trajectory, as well as their values and beliefs regarding quality of life. Important implications of these findings include the need for psychoeducation, individualized meetings with healthcare professionals, and specialist memory clinics to liaise with services that can provide socioemotional support for patients and their caregivers.

## Figures and Tables

**Table 1:** Demographics of participants.

	Gender	Age	Ethnicity	Education	Relationship	Risk of cognitive impairment	Decision
P1	Female	61	Eurasian	16	Daughter	Severe	Refused
P2	Female	60	Chinese	10	Daughter-in-law	Severe	Accepted
P3	Female	54	Chinese	12	Daughter	Severe	Accepted
P4	Female	55	Chinese	10	Daughter	Moderate	Refused
P5	Female	59	Malay	10	Daughter	Severe	Refused

*Note.* Relationship = caregiver's relationship to the patient. Decision = decision regarding diagnostic assessment.

**Table 2:** Master themes and subthemes.

Master themes	Subthemes
Gathering information	Observing the patient
	Making comparisons
Protecting the patient	From distress
	From futility
Balancing obligation and convenience in caregiving	Duty of caring and advocating
	Easing caregiver burden

## Data Availability

The data used to support the findings of this study are included within the article.
